# Predictors of response to intra-arterial vasodilatory therapy of non-occlusive mesenteric ischemia in patients with severe shock: results from a prospective observational study

**DOI:** 10.1186/s13054-022-03962-w

**Published:** 2022-04-04

**Authors:** Nina Rittgerodt, Thorben Pape, Markus Busch, Lena S. Becker, Andrea Schneider, Heiner Wedemeyer, Benjamin Seeliger, Julius Schmidt, Anna Maria Hunkemöller, Jan Fuge, Wolfgang Knitsch, Christine Fegbeutel, Hans-Jörg Gillmann, Bernhard C. Meyer, Marius M. Hoeper, Jan B. Hinrichs, Sascha David, Klaus Stahl

**Affiliations:** 1 Department of Gastroenterology, Hepatology and Endocrinology, Hannover Medical School, Carl-Neuberg-Str.1, 30625 Hannover, Germany; 2 Department of Diagnostic und Interventional Radiology, Hannover Medical School, Hannover, Germany; 3 Department of Respiratory Medicine and German Centre of Lung Research (DZL), Hannover Medical School, Hannover, Germany; 4 Department of Nephrology and Hypertension, Hannover Medical School, Hannover, Germany; 5 Department of General, Visceral and Transplant Surgery, Hannover Medical School, Hannover, Germany; 6 Department of Cardiac, Thoracic, Transplant and Vascular Surgery, Hannover Medical School, Hannover, Germany; 7 Department of Anesthesiology and Intensive Care, Hannover Medical School, Hannover, Germany; 8 Institute of Intensive Care Medicine, University Hospital Zurich, Zurich, Switzerland

**Keywords:** Shock, Intestinal failure, Non-occlusive mesenteric ischemia, Sepsis

## Abstract

**Background:**

Non-occlusive mesenteric ischemia (NOMI) is a life-threatening condition occurring in patients with shock and is characterized by vasoconstriction of the mesenteric arteries leading to intestinal ischemia and multi-organ failure. Although minimal invasive local intra-arterial infusion of vasodilators into the mesenteric circulation has been suggested as a therapeutic option in NOMI, current knowledge is based on retrospective case series and it remains unclear which patients might benefit. Here, we prospectively analyzed predictors of response to intra-arterial therapy in patients with NOMI.

**Methods:**

This is a prospective single-center observational study to analyze improvement of ischemia (indicated by reduction of blood lactate > 2 mmol/l from baseline after 24 h, primary endpoint) and 28-day mortality (key secondary endpoint) in patients with NOMI undergoing intra-arterial vasodilatory therapy. Predictors of response to therapy concerning primary and key secondary endpoint were identified using a) clinical parameters as well as b) data from 2D-perfusion angiography and c) experimental biomarkers of intestinal injury.

**Results:**

A total of 42 patients were included into this study. At inclusion patients had severe shock, indicated by high doses of norepinephrine (NE) (median (interquartile range (IQR)) 0.37 (0.21–0.60) μg/kg/min), elevated lactate concentrations (9.2 (5.2–13) mmol/l) and multi-organ failure. Patients showed a continuous reduction of lactate following intra-arterial prostaglandin infusion (baseline: (9.2 (5.2–13) mmol/l vs. 24 h: 4.4 (2.5–9.1) mmol/l, *p* < 0.001) with 22 patients (52.4%) reaching a lactate reduction > 2 mmol/l at 24 h following intervention. Initial higher lactate concentrations and lower NE doses at baseline were independent predictors of an improvement of ischemia. 28-day mortality was 59% in patients with a reduction of lactate > 2 mmol/l 24 h after inclusion, while it was 85% in all other patients (hazard ratio 0.409; 95% CI, 0.14–0.631, *p* = 0.005).

**Conclusions:**

A reduction of lactate concentrations was observed following implementation of intra-arterial therapy, and lactate reduction was associated with better survival. Our findings concerning outcome predictors in NOMI patients undergoing intra-arterial prostaglandin therapy might help designing a randomized controlled trial to further investigate this therapeutic approach.

*Trial registration* Retrospectively registered on January 22, 2020, at clinicaltrials.gov (REPERFUSE, NCT04235634), https://clinicaltrials.gov/ct2/show/NCT04235634?cond=NOMI&draw=2&rank=1.

**Supplementary Information:**

The online version contains supplementary material available at 10.1186/s13054-022-03962-w.

## Background

Non-occlusive mesenteric ischemia (NOMI) is a life-threatening condition that was first described more than 60 years ago in patients with heart failure [1]. In the meantime, NOMI has been described in all forms of shock, especially in sepsis [2]. The mortality associated with NOMI remains unchanged exceedingly high up to above 90% [3, 4]. NOMI accounts for up to 15% of acute mesenteric ischemia cases [5] and is characterized by functional vasoconstriction of the superior mesenteric artery (SMA) and its smaller branches in the absence of an intraluminal obstruction [2, 4, 5]. Spasm of these mesenteric vessels leads to significantly reduced perfusion of the intestine and consequently, mesenteric ischemia, which may result in transmural necrosis. As a consequence of ischemia, the intestinal barrier function can be severely altered resulting in bacterial translocation [6]. This process triggers a secondary systemic inflammatory response that may result in remote organ failure [7]. NOMI occurs typically in the context of shock, especially following cardiac surgery [1–4], in low output heart failure [1, 2, 4] and a variety of heterogeneous acute critical illnesses all requiring high dose vasopressor therapy such as septic shock [2–4, 8]. Various preexisting comorbidities, i.e., heart diseases, chronic or acute kidney disease but also older age and diabetes mellitus increase the risk of developing NOMI [2, 4, 8, 9]. Since emergency surgical intestinal resection has repeatedly shown poor survival and even tends to worsen the underlying pathological processes of NOMI [4, 9], current treatment recommendations aim at rapid re-establishment of intestinal perfusion including fluid resuscitation and reduction of vasoconstrictors [10]. However, reduction of vasopressor dose is often not feasible in situations of profound shock, and fluid resuscitation does not directly affect underlying mesenteric vasoconstriction. In contrast, minimally invasive local intra-arterial infusion of potent vasodilators into the mesenteric circulation via angiographic cannulation was repeatedly shown to be feasible and effective in counteracting mesenteric vasoconstriction [4, 8, 9], without causing unwanted systemic vascular effects. Although intra-arterial therapy has been established as an interventional therapeutic option in NOMI [10], recommendations are mainly based on small retrospective case series. Importantly, no prospective data exist evaluating criteria that might predict response to local intra-arterial therapy of NOMI.

In this prospective observational study (REPERFUSE) we report clinical outcomes of 42 patients with NOMI undergoing local intra-arterial vasodilatory therapy. The present investigation aimed at identifying predictors of response to therapy concerning improvement of ischemia (primary endpoint) as well as 28-day mortality (key secondary endpoint). For this purpose a) clinical factors as well as b) data from 2D-perfusion angiography and c) experimental biomarkers of intestinal injury are analyzed.

## Materials and methods

### Screening and inclusion into the study

This was a prospective, observational, monocentric study investigating outcomes following local intra-arterial prostaglandin therapy in critically ill patients diagnosed with NOMI. The study was conducted in a tertiary care hospital from October 2018 to October 2021. Patients were screened by the medical staff of eight different in house intensive care units (ICUs) for potential existence of NOMI if they fulfilled the following pre-determined inclusion criteria: 1) persistent shock: norepinephrine dose > 0.2 μg/kg/min over > 48 h AND 2) intestinal failure: paralytic ileus > 24 h despite prokinetic therapy AND 3) new onset of progressive organ failure (≥ 2 out of six following criteria): increase in vasopressor dose, rise in serum lactate, decrease in Horowitz index, new need for renal replacement therapy, rise in bilirubin, rise in international normalized ratio (INR), or all of the following: rise in alanine-amino-transferase (ALT), aspartate-amino-transferase (AST), creatine kinase (CK) and lactate dehydrogenase (LDH). Exclusion criteria were age < 18 years and pregnancy. If patients met inclusion criteria, a standardized diagnostic workup following an in house protocol (Additional file 1: Fig. 1) was initiated employing initial biphasic contrast enhanced computed tomography angiography (CT-A) followed by digital subtraction angiography (DSA). Images were acquired using a 64-row scanner (GE LightspeedVCT, GE-Healthcare, Chalfont St.Giles, United Kingdom) or a dual-source CT (Somatom Force, Siemens, Forchheim, Germany) with a reconstructed slice thickness of 1 mm. The imaging protocol consisted of an arterial and venous phase of the entire abdomen with threshold based bolus triggering in the aorta. The original radiographic report on CT imaging was independently reviewed by an experienced radiologist. If NOMI was diagnosed and no signs of advanced intestinal necrosis (e.g., free abdominal fluid, pneumatosis intestinalis, portal venous gas) were present, patients were included into the study and intra-arterial vasodilatory therapy was initiated immediately. Prior to final study inclusion, informed consent was obtained from each participant or her/his legal representative. The study was performed in accordance with the ethical standards laid down in the 1964 Declaration of Helsinki and its later amendments and approved by the local ethical committee (No. 8092_BO_S_2018). The study was registered at clinicaltrials.gov (REPERFUSE, NCT04235634).

**Fig. 1:**
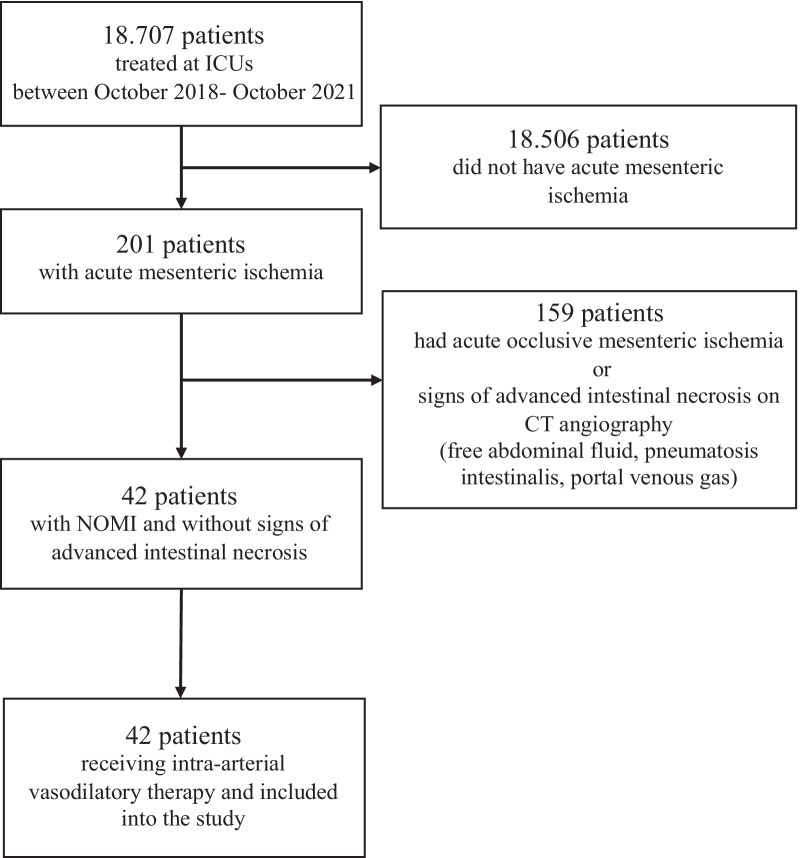
Flowchart of study participants. Flowchart demonstrates inclusion of patients into the observational study. *ICU* intensive care unit, *CT* computed tomography, *NOMI* non-occlusive mesenteric ischemia

### Angiography and local intra-arterial vasodilatory therapy

Vascular access was achieved through the common femoral artery and a 4 French hemostasis sheath (Avanti + , Cordis, Miami Lakes, Florida, USA) was placed. A diagnostic catheter (Radifocus®, Glidecath Cobra2, Terumo Europe, Leuven, Belgium) was advanced in the SMA. Angiography was obtained to verify the correct catheter position. A bolus of 20 μg of prostaglandin E1 (Alprostadil, UCB Pharma GmbH, Monheim, Germany) was slowly infused in the SMA over 10 min. Subsequently, another angiography documented the early treatment response. The sheath and the catheters were fixed, labeled and attached to a continuous infusion drip of prostaglandin at a dose of 60–80 μg/24 h depending on patient weight and following previous reported dosing instructions [11–13]. The duration of prostaglandin infusion was based on the individual course and continued for at least 24 h and as long as feasible if clinical improvement was achieved. Clinical improvement was defined as a combination of clinical observations determined by the primarily treating intensivist. Among these criteria of clinical improvement were hemodynamic improvement (indicated by significant reduction of norepinephrine dose compared to baseline), improvement of organ dysfunction (indicated by any reduction of SOFA score), improvement of bowel ischemia (indicated by a significant reduction of lactate concentrations) and resolution of paralytic ileus (indicated by regular bowel movements). Treatment was stopped on individualized decision of the intensive care team if no treatment response was achieved or patient deceased.

### End points

The aim of the study was to identify predictors of response to intra-arterial therapy concerning improvement of ischemia as well as survival. The primary endpoint was improvement of ischemia defined as a reduction of blood lactate concentration > 2 mmol/l at 24 h following start of intra-arterial vasodilator infusion. The key secondary endpoint was 28-day mortality. Further secondary endpoints were dose of vasopressor support and degree of organ dysfunction, as indicated by the SOFA score, both at 24 h following study inclusion. As potential predictors of treatment response were analyzed: a) routine clinical parameters at inclusion and 24 h after inclusion (available from all patients), b) data from 2D-perfusion angiography at angiographic intervention (from *n* = 30 patients) and c) biomarkers of intestinal ischemia at inclusion (from *n* = 22 patients). These parameters were then stratified for patients with and without an improvement of ischemia 24 h following start of intervention as well as for survivors and non-survivors, respectively.

### Data collection

Routine clinical data were collected at study inclusion and 24 h following study inclusion using electronic medical records including the patient data monitoring system (PDMS) m-life. SOFA scores were calculated according to the description by Vincent et al. [14]. Organ failure was defined as an organ specific SOFA score of equal or greater than two.

### 2D-perfusion angiography

Post-processing of DSA runs was performed on a dedicated workstation (syngo X Workplace® VD20D, Siemens Healthcare) and two radiologists (J.B.H., L.S.B.) agreed upon region of interest (ROI) placement. A reference ROI was fitted to at least two-thirds of the vessel diameter and placed in the SMA next to the tip of the inserted diagnostic catheter and therewith at the location of CM influx. One target ROI was placed in the main stem of the portal vein (ROI-PV), proximal to the bifurcation. A second target ROI was placed in the aorta (ROI-Aorta) close to the origin of the SMA to detect contrast reflux at its origin. Numeric density values for time to peak (TTP), peak density (PD) and the area under the time–density curve (AUC) were recorded. The ratios of the reference to the target ROI, i.e., TTP-PV/TTP-Ref, PD-PV/PD-Ref, AUC-PV/AUC-Ref, TTP-Aorta/TTP-Ref, PD-Aorta/PD-Ref and AUC-Aorta/AUC-Ref before and after vasodilatory therapy were calculated. PD is defined as the maximum density in the chosen ROI after contrast administration, TTP is characterized as the time from the beginning of the angiographic run to the maximum density within the ROI, and AUC visualizes the density values within the ROI during the span of an angiographic run [15]. Shorter TTP-PV as well as higher PD-PV and AUC-PV would indicate better portal vein filling as a surrogate of better mesenteric perfusion. Moreover, longer TTP-Aorta as well as lower PD-Aorta and AUC-Aorta would indicate less aortic reflux as a surrogate of better mesenteric perfusion. In addition, we assessed a previously published NOMI score [16, 17] that is comprised of five subjectively assessed categories: vessel morphology, aortal contrast reflux, contrast enhancement of the intestine, distension of the intestine and time to portal vein filling. The score was calculated before and directly after intervention and ranges from 0–11 points, with higher scores indicating more severe angiographic changes characteristic of NOMI. NOMI score was assessed in 40 patients and parameters from 2D-perfusion angiography in 30 patients.

### ELISA measurements

Plasma was taken at inclusion directly before the intervention (*n* = 22 patients) as well as from individuals without any acute illness controls (*n* = 20). The EDTA plasma samples were centrifuged at 4 °C and 3800 rpm for 10 min and stored at − 20 °C. Due to various different ICU teams and organizational heterogeneities the blood was taken only in a subset of patients (*n* = 22). Intestinal fatty-acid binding protein (i-FABP) and liver fatty-acid binding protein (L-FABP) are found increased in mucosal intestinal injury [18]. Smooth muscle protein 22 (SM22) is a potential plasma marker to detect severe transmural intestinal [7, 19]. Enzyme-linked immunosorbent assays (ELISA) were performed for L-FABP and i-FABP from human plasma using the commercially available L-FABP (HK404-01, HycultBiotech, Uden, The Netherlands) and i-FABP kit (HK406-01, HycultBiotech, Uden, The Netherlands) assays, respectively. For L-FABP plasma was diluted 1:20; for i-FABP plasma was diluted 1:2. SM22 was measured in human plasma by commercial human TAGLN/Transgelin/SM22 ELISA kit (LS-F7946, LifeSpan BioSciences, Inc., Seattle, WA, USA). For SM22 ELISA, human plasma was used undiluted. All ELISAs were performed according to manufacturer’s instructions.

### Statistical analysis

We used GraphPad Prism 7 (La Jolla, CA), IBM SPSS Statistics (version 27 IBM Corp., Armonk, NY) and STATA (version 13.0, StataCorp, College Station, TX) for data analysis and graph generation. Categorical variables are shown as numbers (*n*) and percentages (%). Continuous variables are shown as median and 25%-75% quartiles, unless indicated otherwise. Variables were checked for normal distribution using the D’Agostino-Pearson omnibus normality test and the Shapiro–Wilk normality test. For comparison of categorical variables Chi-squared test was used. For paired comparison of continuous variables two-sided paired t-test and Wilcoxon matched-pairs signed rank test were used for normally and non-normally distributed variables, respectively. For unpaired comparison of continuous variables two-sided unpaired t-test and Mann–Whitney test were used for normally and non-normally distributed variables, respectively. For comparison of lactate concentrations at multiple time points before and after start of intra-arterial therapy one-way repeated measures ANOVA was used. Univariate and multivariate logistic regressions were conducted for the primary endpoint. All variables reaching statistical significance in the univariate regression analysis were subsequentially entered into a forward-step conditional multivariate model. Variables were excluded from the multivariate model if they had a *p*-value that was not below 0.1 and were therefore considered to be not beneficial for explanation of the dependent outcome variable. Survival data were analyzed by log-rank test as well as by Cox-regression analysis and were visualized by Kaplan–Meier curves. All reported *p*-values are two-sided unless indicated otherwise; *p*-values < 0.05 were considered statistically significant.

## Results

### Cohort characterization

From October 2018 to October 2021, a total of 201 patients treated at 11 ICUs were diagnosed with acute mesenteric ischemia. After exclusion of patients with occlusive mesenteric ischemia and patients with characteristics of advanced intestinal necrosis on CT imaging, 42 patients with potentially reversible NOMI received intra-arterial vasodilatory therapy and were included into the study (Fig. 1). Demographic and clinical parameters at study inclusion are demonstrated in Table 1. Most common comorbidities were hypertension, obesity, coronary artery disease (CAD) and chronic kidney disease (CKD). All patients were diagnosed with sepsis. Ninety-five percent of patients had refractory septic shock indicated by high doses of norepinephrine (NE) (median (interquartile range (IQR)) 0.368 (0.212–0.598) μg/kg/min) and severely elevated lactate concentrations (9.2 (5.2–13) mmol/l). All patients suffered from multi-organ failure with 83% presenting with five or six organ failure at inclusion. Seventy-six percent of patients were invasively ventilated, 83% received renal replacement therapy (RRT) and the median SOFA score was 17. Routine laboratory parameters that have been associated with NOMI previously (e.g., lactate, CK, LDH, AST, bilirubin) [2, 8, 10, 20] were all significantly elevated before initiation of intra-arterial therapy. Included patients received angiography with initial classification of NOMI morphological severity. The median NOMI score at inclusion was 5 (4–7) points. All patients received a PGE_1_ bolus, followed by continuous infusion of PGE_1_ for a total of 57 (28–99) hours. The angiographic cannulation as well as the prostaglandin infusions were found to be safe with no apparent procedure-related or drug-related adverse events.

**Table 1:** Demographic and clinical characteristics at study inclusion

Category	Median (IQR)/No (%)
	(n = 42)
Age, year	61 (48–70)
Sex, no (%)	
Male	22 (52.4)
Female	20 (47.6)
BMI, kg/m^2^	27.7 (23.3–29.8)
Comorbidities, no (%)	
Obesity	15 (35.7)
Hypertension	24 (57.1)
Diabetes	8 (19)
COPD	4 (9.5)
Heart insufficiency	11 (26.2)
CAD	16 (38.1)
CABG	4 (9.5)
PTCA	10 (23.8)
CKD	15 (35.7)
Chronic renal replacement therapy	10 (23.8)
Immunosuppression	3 (7.1)
Sepsis, no (%)	42 (100)
Side of infection, no (%)	
Pulmo	23 (54.8)
Abdomen	33 (78.6)
Urogenital	8 (19)
Soft tissue	11 (26.2)
Endocarditis	3 (7.1)
More than one	27 (64.3)
Identified pathogen, no (%)	
Gram +	15 (35.7)
Gram −	20 (47.6)
Viral	3 (7.1)
Fungi	11 (26.2)
More than one	13 (31)
Non identified	11 (26.2)
Renal replacement therapy, no (%)	32 (76.2)
Invasive ventilation, no (%)	35 (83.3)
Oxygenation index (PaO_2_/FiO_2_)	200 (134–286)
SOFA score, points	17 (15–19)
Norepinephrine, no (%)	40 (95.2)
Norepinephrine dose, μg/kg/min	0.368 (0.212–0.598)
Argipressin, no (%)	9 (21.4)
Dobutamine, no (%)	4 (9.5)
Organ failure, no (%)	
Respiratory (PaO_2_/FiO_2_ < 300 mmHg)	39 (92.9)
Coagulation (Thrombocytes < 100^3^/µl)	32 (76.2)
Liver (Bilirubin > 33 μmol/l)	32 (76.2)
Cardiovascular (vasopressor or inotrope)	40 (95.2)
Neurological (GCS < 13)	35 (83.3)
Renal (Creatinine > 170 μmol/l)	37 (88.1)
Multi-organ failure, no (%)	
2	0 (0)
3	4 (9.5)
4	2 (4.8)
5	16 (38.1)
6	19 (45.2)
pH	7.27 (7.18–7.36)
Bicarbonate, mmol/l	20 (16–22)
Lactate, mmol/l	9.2 (5.2–13)
CK, IU/l	1748 (332–5400)
LDH, U/l	1174 (526–3877)
AST, U/l	267 (143–1422)
ALT, U/l	127 (57–390)
Bilirubin, µmol/l	78 (30.5–158)
Leucocytes, 1000/µl	10.7 (7.9–21.2)
CRP, mg/l	108 (44–182)
PCT, µg/l	5.7 (1.8–23.6)
Thrombocytes, 100^3^/µl	69 (34.5–141)
Simplified NOMI score, points	5 (4–7)

*ALT* Alanine aminotransferase, *ATIII* antithrombin III, *AUC* area under the curve, *AST* aspartate aminotransferase, *BMI* body mass index, *CAD* coronary artery disease, *CK* creatine kinase, *CKD* chronic kidney disease, *COPD* chronic obstructive pulmonary disease, *CRP* C-reactive protein, *GCS* Glasgow Coma scale, *LDH* lactate dehydrogenase, *NE* norepinephrine, *PCT* procalcitonine, *PTCA* percutaneous transluminal coronary angioplasty, *SOFA* Sequential Organ Failure Assessment

### Clinical endpoints

**Fig. 2:**
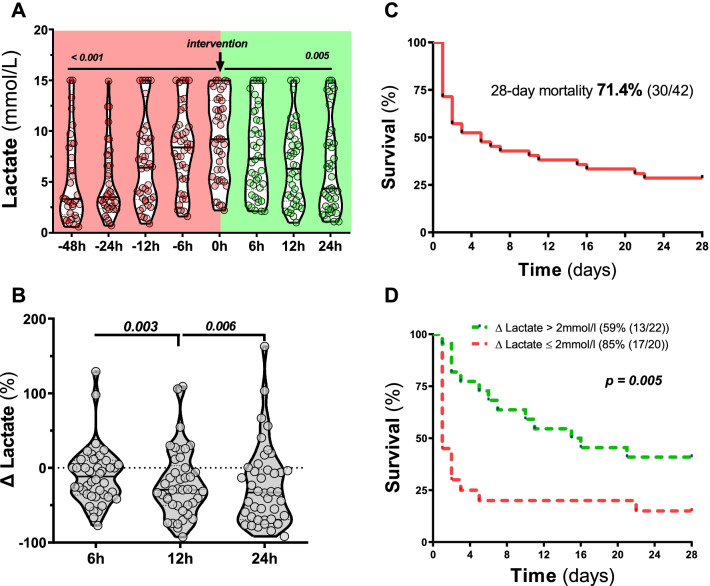
Primary and key secondary outcomes. Kaplan–Meier graphs showing the 28-day survival course in the overall cohort (primary outcome (**A**) and in patients stratified for lactate reduction > 2 mmol/l (**B**). Violine plots showing time course of lactate concentration 48, 24, 12 and 6 h before inclusion, at inclusion as well as after 6, 12 and 24 h (key secondary outcome) following inclusion (**C**). Violine plots demonstrating reduction of lactate concentration in relation to baseline at 6, 12 and 24 h following inclusion (**D**)

Lactate concentrations continuously increased within the first 48 h before begin of intra-arterial prostaglandin infusion (lactate at 48 h before inclusion: (3.3 (1.8–8.6) mmol/l vs. at inclusion: (9.2 (5.2–13) mmol/l (*p* = 0.001), overall from 48 h before to study inclusion: *p* < 0.001, Fig. 2A). With intra-arterial prostaglandin therapy, lactate levels declined (lactate at inclusion vs. 6 h following intervention: 7.3 (4.2–11.3) mmol/l (*p* = 0.01), vs. 12 h: 6.3 (3.1–9.3) mmol/l (*p* < 0.001), vs. 24 h: 4.4 (2.5–9.1) mmol/l (*p* < 0.001), overall from inclusion to 24 h after inclusion: *p* = 0.005, Fig. 2A). This corresponded to a relative reduction of lactate concentrations of − 12% (-36 to + 10) at 6 h, − 29% (-50 to + 3) at 12 h and − 33% (-69 to − 2) at 24 h (overall *p* < 0.001) compared to baseline lactate (Fig. 2B). Thirty-two (76%) of patients showed no further increase of lactate. Twenty-two (52%) patients had an improvement of ischemia as indicated by a reduction of lactate > 2 mmol/l within 24 h (primary outcome).

The overall 28-day in-hospital mortality (key secondary outcome) was 71% (Fig. 2C). 28-day mortality was 59% in patients, who experienced a reduction of lactate > 2 mmol/l within 24 h, while it was 85% in all other patients (HR 0.409 (0.14–0.631), *p* = 0.005, Fig. 2D).

SOFA scores (*p* = 0.569) and norepinephrine doses (*p* = 0.667) were unchanged at 24 h after inclusion compared to baseline (results not shown). Median (IQR) NOMI score significantly decreased following initial prostaglandin administration indicating improvement of intestinal perfusion judged by five different semi-quantitative categories (Additional file 2: Fig. 2A). Peak density in the portal vein (PD-PV) increased (Additional file 2: Fig. 2B) and time to peak (TTP-PV) decreased (Additional file 2: Fig. 2C) following initial prostaglandin bolus, indicating better and faster portal vein filling, whereas reduced area under the curve contrast intensity in the aorta (AUC-Aorta) suggested less reflux into the aorta after vasodilator infusion (Additional file 2: Fig. 2D). Non-routine biomarkers of intestinal ischemia were all significantly increased at study inclusion compared to controls (Additional file 3: Fig. 3).

### Predictors of primary and key secondary endpoint

As potential predictors of primary (improvement of ischemia (reduction lactate > 2 mmol/l at 24 h)) and key secondary outcome (28-day mortality), we analyzed: a) routine clinical parameters at inclusion and 24 h after inclusion, b) data from 2D-perfusion angiography at angiographic intervention and c) experimental biomarkers of intestinal ischemia at inclusion (Tables 2, 3 and Additional file 4: Table 1).

**Table 2:** Demographic, clinical, angiographic and biochemical characteristics for patients with and without improvement of ischemia (lactate reduction > 2 mmol/l 24 h after intervention)

Category	No improvement of ischemia (n = 20)	Improvement of ischemia (n = 22)	*p*
Age, year	66 (53–73)	55 (45–68)	0.432
Sex, no (%)			0.118
Male	13 (65.0)	9 (40.9)	
Female	7 (35.0)	13 (59.1)	
BMI, kg/m^2^	26.3 (21.5–29.6)	27.7 (24,4–30,3)	0.331
Comorbidities, no (%)			
Obesity	7 (35.0)	8 (36.4)	0.927
Hypertension	13 (65.0)	11 (50.0)	0.327
Diabetes	6 (30.0)	2 (9.1)	0.085
COPD	3 (15.0)	1 (4.5)	0.249
Heart insufficiency	5 (45.5)	6 (54.5)	0.867
CAD	9 (45.0)	7 (31.8)	0.380
CABG	3 (15.0)	1 (4.5)	0.249
PTCA	5 (25.0)	5 (22.7)	0.863
CKD	10 (50.0)	5 (22.7)	0.065
Chronic renal replacement therapy	7 (35.0)	3 (13.6)	0.104
Immunosuppression	3 (15.0)	0 (0)	0.059
Sepsis, no (%)	20 (100)	22 (100)	–
Side of infection, no (%)			
Pulmo	11 (55.0)	12 (54.5)	0.976
Abdomen	15 (75.0)	18 (81.8)	0.591
Urogenital	5 (25.0)	3 (13.6)	0.349
Soft tissue	5 (25.0)	6 (27.3)	0.867
Endocarditis	0 (0)	3 (13.6)	0.087
More than one	13 (65.0)	14 (63.6)	0.927
Identified pathogen, no (%)			
Gram+	3 (15.0)	12 (54.5)	**0.008**
Gram−	9 (45.0)	11 (50.0)	0.746
Viral	1 (5.0)	2 (9.1)	0.607
Fungi	6 (30.0)	5 (22.7)	0.592
More than one	5 (25.0)	8 (36.4)	0.426
Non identified	6 (30.0)	5 (22.7)	0.592
**At inclusion**			
SOFA score, points	17 (16–19)	18 (13.5–19)	0.319
Norepinephrine, no (%)	19 (95.0)	21 (95.5)	0.945
Norepinephrine dose, µg/kg/min	0.52 (0.26–0.79)	0.34 (0.18–0.47)	**0.021**
Argipressin, no (%)	4 (20.0)	5 (22.7)	0.830
Dobutamine, no (%)	2 (10.0)	2 (9.1)	0.920
Invasive ventilation, no (%)	17 (85.0)	18 (81.8)	0.782
Oxygenation index (PaO_2_/FiO_2_)	197 (142–334)	200 (111–271)	0.429
Renal replacement therapy, no (%)	16 (80.0)	16 (50.0)	0.580
Organ failure, no (%)			
respiratory (PaO_2_/FiO_2_ < 300 mmHg)	19 (95.0)	20 (90.9)	0.607
Coagulation (thrombocytes < 10^3^/µl)	17 (85.0)	15 (68.2)	0.201
Liver (Bilirubin > 33 μmol/l)	15 (75.0)	17 (77.3)	0.863
Cardiovascular (vasopressor or inotrope)	19 (95.0)	21 (95.5)	0.945
Neurological (GCS < 13)	18 (90.0)	17 (77.3)	0.269
Renal (Creatinine > 170 μmol/l)	18 (90.0)	19 (86.4)	0.716
Multi-organ failure, no (%)			
2	0 (0)	0 (0)	1
3	1 (5.0)	3 (13.6)	0.341
4	1 (5.0)	1 (4.5)	0.945
5	9 (45.0)	7 (31.8)	0.380
6	9 (45.0)	10 (45.5)	0.976
pH	7.27 (7.18–7.37)	7.27 (7.17–7.34)	0.606
Bicarbonate, mmol/l	21.5 (19.0–22.0)	18.0 (15.8–21.8)	0.190
Lactate, mmol/l	5.9 (3.4–11.7)	11.4 (8.1–13.5)	**0.01**
CK, U/l	1250 (375–2596)	2226 (332–6464)	0.742
LDH, U/l	823 (499–3722)	1305 (505–4658)	0.410
AST, U/l	240 (143–940)	427 (138–3570)	0.172
ALT, U/l	121 (49–294)	136 (58–436)	0.350
Bilirubin, µmol/l	66 (24–134)	85 (49–172)	0.376
Leucocytes, 1000/µl	8.9 (4.8–20.8)	14.5 (10.1–21.7))	0.449
CRP, mg/l	114 (58–247)	108 (38–159)	0.399
PCT, µg/l	5.9 (2.1–42.7)	5.4 (1.7–20.7)	0.421
Thrombocytes, 100^3^/µl	51.0 (22.3–108.8)	71.0 (46.4–158.0)	0.268
INR	1.53 (1.18–1.94)	1.62 (1.36–2.06)	0.172
PTT, sec	50.0 (42.3–70.8)	55 (46–63)	0.856
ATIII, mg/dl	51 (35–56)	45 (37–61)	0.967
**After 24 h**			
SOFA score, points	17 (16–20)	17 (14–19)	0.219
Norepinephrine, no (%)	19 (95.0)	19 (86.4)	0.341
Norepinephrine dose, µg/kg/min	0.45 (0.26–0.93)	0.19 (0.05–0.49)	**0.009**
Argipressin, no (%)	4 (20.0)	4 (18.2)	0.881
Dobutamine, no (%)	2 (10.0)	3 (13.6)	0.716
Invasive ventilation, no (%)	17 (85.0)	18 (81.8)	0.782
Oxygenation index (PaO_2_/FiO_2_)	236.0 (109.8–294.5)	251.0 (156.0–348.0)	0.314
Organ failure, no (%)			
Respiratory (PaO_2_/FiO_2_ < 300 mmHg)	18 (90.0)	18 (81.8)	0.449
Coagulation (Thrombocytes < 10^3^/µl)	18 (90.0)	19 (90.5)	0.959
Liver (Bilirubin > 33 μmol/l)	16 (80.0)	17 (81.0)	0.939
Cardiovascular (vasopressor or inotrope)	19 (95.0)	20 (90.9)	0.607
Neurological (GCS < 13)	17 (85.0)	17 (77.3)	0.524
Renal (Creatinine > 170 μmol/l)	18 (90.0)	19 (86.4)	0.716
Multi-organ failure, no (%)			
2	0 (0)	2 (9.1)	0.167
3	1 (5.0)	0 (0)	0.288
4	2 (10.0)	2 (9.1)	0.920
5	7 (35.0)	4 (18.2)	0.216
6	10 (50.0)	13 (59.1)	0.554
CK, U/l	1649.0 (672.5–10,904.0)	1412.0 (409.5–8087.5)	0.729
LDH, U/l	875.0 (329.3–7445.3)	1345.0 (518.5–3389.5)	0.746
AST U/l	809.0 (261.8–4167.8)	1116.0 (167.5–2850.0)	0.590
ALT U/l	328.0 (96.0–2169.5)	214.0 (126.0–496.5)	0.510
Bilirubin, µmol/l	77.0 (40.0–125.0)	60.5 (36.8–157.5)	0.709
Leucocytes, 1000/µl	12.1 (6.2–19.3)	13.4 (9.5–20.6)	0.818
CRP, mg/l	105.5 (65.8–241.0)	108.5 (31.8–194.8)	0.624
PCT, µg/l	6.4 (1.0–22.7)	4.9 (2.9–22.3)	0.726
Thrombocytes, 1000/µl	60.0 (29.0–105.0)	70 (32–110)	0.777
**2D-perfusion angiography (directly pre-vasodilator)**			
Simplified NOMI score	5 (4–6)	5 (4–7)	0.767
PD-PV	0.78 (0.27–1.39)	0.62 (0.525–1–59)	0.997
TTP-PV, sec	11.68 (9.86–12.85)	11.3 (9.9–14.0)	0.749
AUC-PV	0.57 (0.28–1.30)	0.55 (0.37–1.23)	0.767
PD-Aorta	0.9 (0.37–2.54)	0.57 (0.27–1.93)	0.916
TTP-Aorta, sec	6.43 (5.37–9.34)	8.1 (6.1–9.7)	0.474
AUC-Aorta	0.74 (0.56–2.56)	0.56 (0.27–2.15)	0.703
**2D-perfusion angiography (directly post-vasodilator)**			
Simplified NOMI score	3 (1–3)	2 (1–3)	0.767
PD-PV	1.32 (0.39–1.97)	0.8 (0.4–1.4)	0.189
TTP-PV, sec	10.7 (9.0–12.2)	10.3 (7.9–11.5)	0.570
AUC-PV	0.85 (0.43–1.08)	0.64 (0.27–0.81)	0.195
PD-Aorta	0.55 (0.35–2.19)	0.39 (0.22–1.06)	0.178
TTP-Aorta, sec	6.43 (5.81–8.12)	8.66 (7.79–11.0)	**0.043**
AUC-Aorta	0.61 (0.33–2.44)	0.34 (0.23–0.76)	0.054
Duration PGE_1_, hours	30.0 (21.0–69.5)	79.0 (50.4–141.0)	**0.001**

*ALT* Alanine aminotransferase, *ATIII* Antithrombin III, *AUC* Area under the curve, *AST* Aspartate aminotransferase, *BMI* Body mass index, *CAD* Coronary artery disease, *CK* creatine kinase, *CKD* Chronic kidney disease, *COPD* Chronic obstructive pulmonary disease, *CRP* C-reactive protein, *GCS* Glasgow Coma scale, *i-FABP* intestinal Fatty-acid binding protein, *INR* international normalized ratio, *LDH* Lactate dehydrogenase, *L-FABP* Liver fatty-acid binding protein, *NE* Norepinephrine, *PD* Peak density, *PCT* Procalcitonine, *PTCA* Percutaneous transluminal coronary angioplasty, *PTT* Partial thromboplastin time, *PV* Portal vein, *SM22* Smooth muscle protein 22, *SOFA* Sequential Organ Failure Assessment, *TTP* Time to peak

**Table 3:** Predictors of primary outcome: improvement of ischemia (lactate reduction > 2 mmol/l 24 h after intervention)

Characteristic		Univariate			Multivariate	
	OR	95% CI	*p*	OR	95% CI	*p*
Norepinephrine dose, 0.1 µg/kg/min	0.759	0.591–0.977	0.032	0.551	0.329–0.942	0.024
Lactate, 1 mmol/l	1.243	1.046–1.478	0.014	1.519	1.031–2.238	0.034
TTP-Aorta post bolus, 1 s	1.377	0.978–1.941	0.067			

*TTP* time to peak

Patients with (*n* = 22) and without (*n* = 20) an improvement of ischemia were comparable in most demographic and clinical characteristics at baseline (Table 2). However, patients with improvement of ischemia had lower NE doses at inclusion (*p* = 0.021) and 24 h after inclusion (*p* = 0.009). Lactate concentrations were almost twice as high in patients that significantly reduced lactate 24 h following start of prostaglandin infusion (*p* = 0.01). NOMI Scores and most parameters of 2D perfusion angiography were comparable between both groups. Still, higher AUC (*p* = 0.054) and longer time to contrast peak (*p* = 0.043) in the aorta could be found in patients with a significant lactate reduction after vasodilator administration. Patients with an improvement of ischemia received intra-arterial infusion significantly longer (*p* = 0.001). On univariate regression analysis higher lactate concentrations and lower NE doses at inclusion as well as longer TTP-Aorta post bolus were associated with improvement of ischemia (Table 3). On multivariate regression analysis only higher lactate concentration and lower NE doses at baseline were independently associated with significant lactate reduction following 24 h of prostaglandin therapy (Table 3).

Demographic and clinical parameters for survivors (*n* = 12) and non-survivors (*n* = 30) are shown in Additional file 4: Table 1. Thrombocytes were lower in non-survivors both at inclusion and at 24 h after inclusion (*p* = 0.024). Non-survivors showed higher INR at inclusion (*p* = 0.017). This corresponded to higher coagulation specific SOFA sub-scores at both inclusion (*p* = 0.003) and 24 h (*p* = 0.032) after inclusion in non-survivors. While NE dose decreased in survivors, it further increased in non-survivors at 24 h (*p* < 0.001). 24 h following start of intra-arterial therapy, lactate (*p* = 0.002) was significantly higher, whereas bicarbonate (*p* = 0.001) and pH (*p* = 0.034) were significantly lower in later non-surviving patients. I-FABP was significantly higher in survivors at inclusion (*p* = 0.04). Due to the small fraction of surviving patients, no additional regression analysis was performed to analyze this endpoint.

## Discussion

In this prospective observational study of 42 critical ill patients with NOMI we investigated outcomes and predictors of response to intra-arterial vasodilatory therapy. In summary, a rapid reduction of lactate concentrations as a surrogate of improvement of ischemia was observed following implementation of intra-arterial therapy and lactate reduction was associated with better survival. Further predictors for response to intra-arterial vasodilatory therapy were investigated including a variety of clinical routine parameters, data from 2D-perfusion angiography and experimental biomarkers of ischemic intestinal injury.

The inclusion criteria of persistent vasopressor-dependent shock, paralytic ileus and new onset organ failure and/or biochemical signs of ischemia were selected for a severely ill patient cohort with profound refractory shock and progressive multi-organ failure. Almost all patients were diagnosed with septic shock, an important risk factor for NOMI [2]. A median NOMI score at inclusion of 5 indicated angiographic features of critical NOMI as a score ≥ 3.5 was found to present a threshold for poor outcomes in such patients [17]. Both cannulation and prostaglandin infusion were tolerated without any apparent procedure-related side effects, which reassures previously made findings [2, 4]. Reduction of NOMI scores, better and faster portal vein filling and reduced aortic reflux, directly following initial prostaglandin bolus, all together suggest improved mesenteric perfusion caused by intra-arterial vasodilator infusion.

A continuous increase of lactate concentrations could be observed in this study that was reversed following implementation of intra-arterial vasodilatory therapy. Although uncontrolled, this observation might suggest a potential causal effect of intra-arterial prostaglandin infusion on this surrogate marker of intestinal ischemia improvement. Higher lactate concentrations and lower NE doses at baseline were independent predictors of an improvement of ischemia. A reduction of aortic reflux (indicated by both lower AUC and longer TTP values for the aorta) detected immediately after initial prostaglandin bolus administration was also observed more commonly in patients with initial improvement of intestinal ischemia. One could speculate that a combination of both a more profound intestinal perfusion deficit (indicated by higher lactate) and a less severe shock (lower NE dose) might dictate the likelihood of initial ischemia improvement, which might be at least partially caused by intra-arterial prostaglandin infusion (lower AUC-Aorta and TTP-Aorta).

28-day mortality was still high despite all patients receiving prostaglandin infusion. The mortality rate of 71% was however comparable to a recent analysis reporting a 30-day mortality of 66% in NOMI patients of comparable disease severity before commencement of intra-arterial infusion using the vasodilator papaverine [21]. In the same retrospective study, matched control patients, receiving standard medical therapy only, had a miserable survival of only 3% [21]. Indeed, a median SOFA score of 17 in this present cohort at inclusion would predict an expected mortality rate of above 90% [22, 23]. Of note, 28-day mortality was 59% in patients who experienced a reduction of lactate > 2 mmol/l within 24 h of intra-arterial therapy, while it was 85% in all other patients, suggesting that a relevant reduction of lactate concentration within the first 24 h following intra-arterial therapy may indicate a better prognosis. Severe coagulopathy, indicated by thrombocytopenia and increased INR, was associated with mortality, an observation that confirmed the results of previous retrospective analyses in NOMI patients [2]. 24 h after the start of the intervention, NE dose and lactate concentration were significantly higher while pH and base excess were significantly lower in later non-surviving patients. This observation demonstrates both the importance of serial evaluation in adequately predicting the risk of inferior outcomes in NOMI patients undergoing vasodilatory therapy but it also suggests that overall severity of disease might be the major factor predicting later survival in the patients. All three here-investigated biomarkers of intestinal injury were highly elevated in NOMI patients compared to controls. Importantly, no significant difference in i-FABP concentrations could be found previously between mucosal and transmural ischemia [19]. Therefore, we additionally tested SM22, a biomarker that has been suggested to discriminate between mucosal injury and more severe transmural ischemia [19]. Patients in the present cohort had significantly higher median SM22 plasma concentrations compared to healthy controls and these levels were within the range that has been previously reported to be associated with histological transmural ischemia [19]. This suggests that at least some of the patients already had transmural injury at inclusion despite the absence of radiologic signs of intestinal necrosis (e.g., free abdominal fluid, pneumatosis intestinalis, portal venous gas). Unrecognized overt intestinal necrosis could clearly have further contributed to the poor survival rate observed. Disappointingly, neither angiographic NOMI scores and various parameters from 2D-perfusion angiography nor markers of intestinal ischemia at inclusion were different between patients with and without improvement of ischemia as well as survivors and non-survivors. However, the key angiographic parameters reported can only describe vasodilatory changes in the mesenteric macro-circulation (e.g., SMA, aorta, PV). As NOMI is known to also involve the smaller mesenteric branches and arcades and presumably even smaller mesenteric vessels [24], namely the mesenteric micro-circulation, it is possible that the given angiographic parameters do not represent the full picture of vasodilatory response to PGE1 within the complete mesenteric vascular compartment. Multiple parameters at inclusion, assessing severity of NOMI associated intestinal ischemia, failed to predict later outcome. This certainly underlies the fact that the prognosis in such critical ill patients with manifest multi-organ failure is only partly dependent on intestinal ischemia itself, but rather overall disease severity itself might be the major contributing factor, influencing outcome [25]. The key pathologic mechanisms involved in the genesis of NOMI represent an exaggerated adequate physiological response to maintain perfusion of vital organs at the expense of mesenteric perfusion in severe shock [24]. It is therefore to expect that intra-arterial treatment with prostaglandin or any other vasodilator may transiently improve mesenteric perfusion but it is not treating the primary problem of shock and inadequate mesenteric circulation to meet metabolic demands [25]. Therefore minimizing splanchnic ischemia is likely to be of temporal benefit making further resuscitation efforts indispensible.

This investigation represents the first and largest prospective observational study on NOMI patients to the present date. The strict inclusion and exclusion criteria as well as a rigorous standardized in house diagnostic algorithm allowed for identification of a relatively homogenous cohort of critical ill NOMI patients without apparent evidence of advanced bowel injury requiring emergency surgery. The hypothesis to combine routine clinical parameters of disease severity with innovative 2D-perfusion angiography imaging and non-routine biomarkers to predict success of a specific intervention targeting NOMI pathophysiology is novel. However, this study has important limitations, mainly its small sample size, the single-center setting and the lack of a control group. Given the uncontrolled design, it is uncertain if intra-arterial prostaglandin therapy is indeed associated with better outcomes in patients with NOMI, both in terms of improvement of ischemia as well as survival. Additionally, 2D-perfusion angiography and bio-sampling were only performed in a subset of patients further limiting the conclusions of this data, especially in terms of more precise prediction of outcomes employing these parameters. Further, early mortality was high potentially confounding interpretation of lactate reduction following implementation of intra-arterial vasodilator therapy.

## Conclusions

A randomized controlled study, enrolling NOMI patients at the earliest time point possible, is needed to test the value of intra-arterial vasodilatory therapy in severe NOMI. This present observational data demonstrating potential factors associated with both improvement of ischemia and increased mortality in patients undergoing this therapy might assist in future planning of such an investigation.

## Supplementary Information

**Additional file 1: Figure 1**. Standardized diagnostic workup for patients with suspected NOMI. If patients met inclusion criteria, a standardized diagnostic workup following an in house protocol was initiated employing initial biphasic contrast enhanced computed tomography angiography (CT) and digital subtraction angiography (DSA). If both examinations suggested presence of NOMI and excluded complications that required emergency surgical exploration a joint decision (Surgery, Critical Care, Interventional Radiology Team) was made to commence on intra-arterial prostaglandin therapy. If patients or their legal representative gave informed consent, patients were included into the study.

**Additional file 2: Figure 2**. Angiographic parameters*.* Violine plots showing analysis of semi-quantitative NOMI score (**A**), peak density (PD-PV) (**B**) and time to peak (TTP-PV) (**C**) in the portal vein as well as area under the curve contrast intensity in the aorta (AU-Aorta) (**E**) in a subset (n = 40 for NOMI score, n = 30 for 2D-perfusion angiography-related parameters) of NOMI patients before (pre) and directly after (post) initial administration of intra-arterial prostaglandin bolus. Median (IQR) NOMI score decreased following initial prostaglandin administration (pre: 5 (4–7) points vs after: 2 (1–3) points, p < 0.001) indicating significant improvement of intestinal perfusion judged by five different categories (vessel morphology, aortal contrast reflux, contrast enhancement of the intestine, distension of the intestine and time to portal vein filling). PD-PV increased (pre: 0.665 (0.5–1.39) vs. after: 1.06 (0.39–1.7), p = 0.071) and TTP-PV decreased (pre: 11.6 (9.9–13.3) sec vs. after: 10.3 (7.9–11.6) sec, p = 0.017) following initial prostaglandin bolus, indicating better and faster portal vein filling. Reduced AUC-Aorta following prostaglandin infusion (pre: 0.64 (0.36–2.16) vs. post: 0.44 (0.26–1.37), p = 0.025) suggests less reflux into the aorta.

**Additional file 3: Figure 3**. Biomarkers of intestinal ischemia*.* Violine plots showing analysis of L-FABP (**A**), i-FABP (**B**) and SM22 (**C**) in NOMI patients (n = 22) at inclusion compared to healthy controls (Ctrl) (n = 20). Median L-FABP concentrations were more than 10 times higher in NOMI patients compared to healthy controls (197 (179–206) ng/ml vs. 16 (10–18) ng/ml, p < 0.0001, **A**), and i-FABP was more than fourfold increased (1990 (671–5186) pg/ml vs. 479 (327–670) pg/ml, p < 0.001, **B**). SM22, a marker of transmural intestinal ischemia, was also significantly increased in NOMI patients (2116 (1971–2439) pg/ml vs. 1402 (1182–1546) pg/ml, p < 0.0001, **C**).

**Additional file 4: Table 1**. Demographic, clinical, angiographic and biochemical characteristics for non-surviving (n = 30) and surviving (n = 12) patients. 28-day mortality was the key secondary outcome. Values are presented as median (25% to 75% interquartile range) or if categorical as numbers and percentages. Peak density (PD), time to peak (TTP) and area under the curve (AUC) are in relation to the superior mesenteric artery (SMA) as reference.

## Data Availability

The datasets used and analyzed are during the current study are available from the corresponding author on reasonable request.

## Abbreviations

ALT: Alanine aminotransferase
ATIII: Antithrombin III
AUC: Area under the curve
AST: Aspartate aminotransferase
BMI: Body mass index
CAD: Coronary artery disease
CK: Creatine kinase
CKD: Chronic kidney disease
CHF: Congestive heart failure
COPD: Chronic obstructive pulmonary disease
CRP: C-reactive protein
ECMO: Extracorporeal membrane oxygenation (vv = venovenous, va = venoarterial)
ELWI: Extravascular lung water index
FFP: Fresh frozen plasma
GCS: Glasgow Coma scale
GEDI: Global end-diastolic index
Hb: Hemoglobin
Hct: Hematocrit
HR: Hazard ratio
ICU: Intensive care unit
i-FABP: Intestinal fatty-acid binding protein
INR: International normalized ratio
L-FABP: Liver fatty-acid binding protein
LDH: Lactate dehydrogenase
MAP: Mean arterial pressure
NE: Norepinephrine
NOMI: Non-occlusive mesenteric ischemia
OR: Odds ratio
PCT: Procalcitonine
PD: Peak density
PGE_1_: Prostaglandin E_1_
PTCA: Percutaneous transluminal coronary angioplasty
PTT: Partial thromboplastin time
PV: Portal vein
RCT: Randomized controlled trial
ROI: Region of interest
RRT: Renal replacement therapy
SMA: Superior mesenteric artery
SM22: Smooth muscle protein 22
SOFA: Sequential organ failure assessment
TTP: Time to peak
WBC: White blood cell count
