# Supplementary figures and images for: Predictors of response to intra-arterial vasodilatory therapy of non-occlusive mesenteric ischemia in patients with severe shock: results from a prospective observational study
Source: Crit Care. 2022 Apr 4;26:92. doi: 10.1186/s13054-022-03962-w (PMC8981621; doi:10.1186/s13054-022-03962-w)

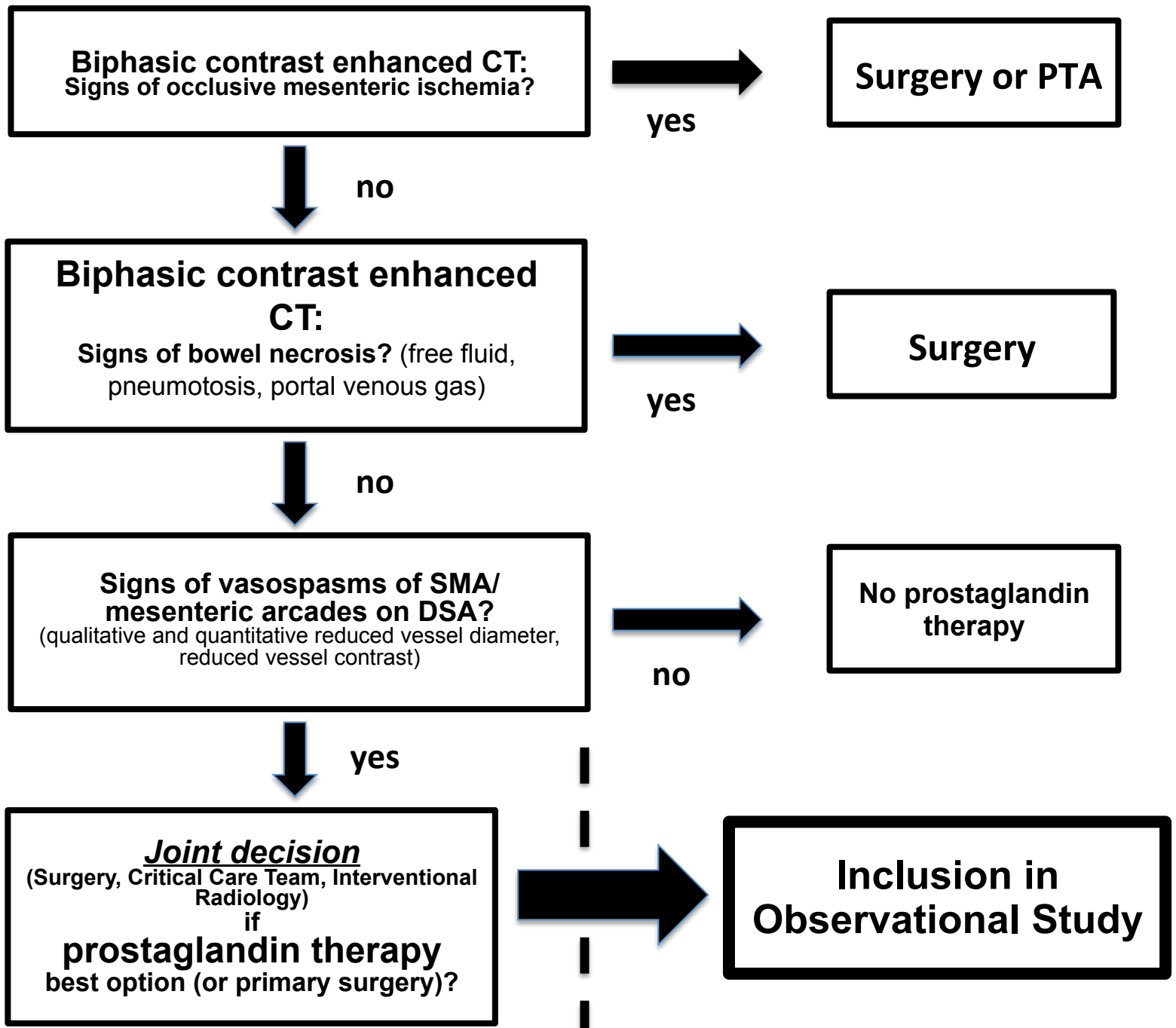

Supplement: Supplementary file 1 — Additional file 1: Figure 1. Standardized diagnostic workup for patients with suspected NOMI. If patients met inclusion criteria, a standardized diagnostic workup following an in house protocol was initiated employing initial biphasic contrast enhanced computed tomography angiography (CT) and digital subtraction angiography (DSA). If both examinations suggested presence of NOMI and excluded complications that required emergency surgical exploration a joint decision (Surgery, Critical Care, Interventional Radiology Team) was made to commence on intra-arterial prostaglandin therapy. If patients or their legal representative gave informed consent, patients were included into the study. [file 13054_2022_3962_MOESM1_ESM.pdf]

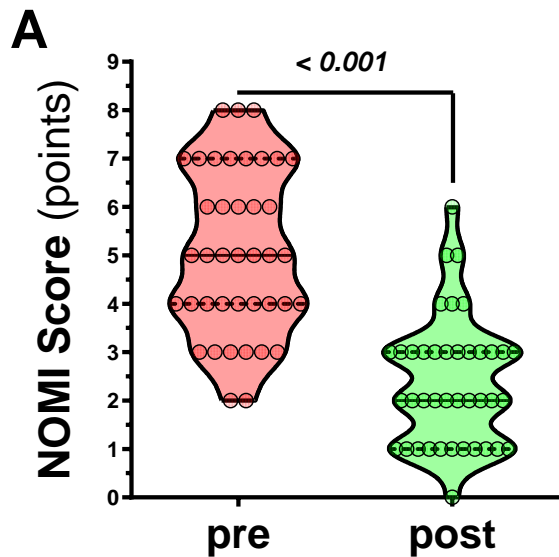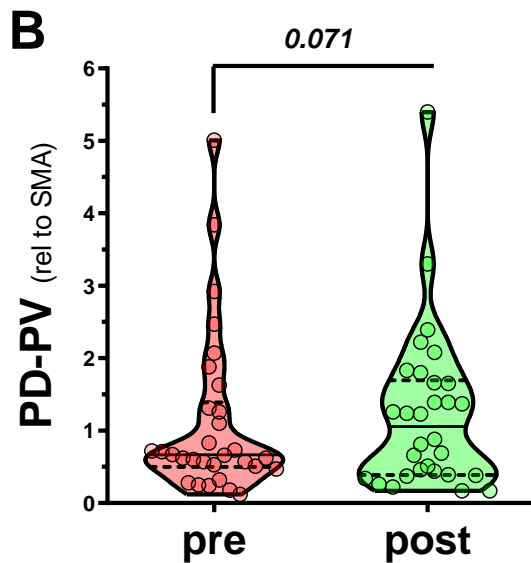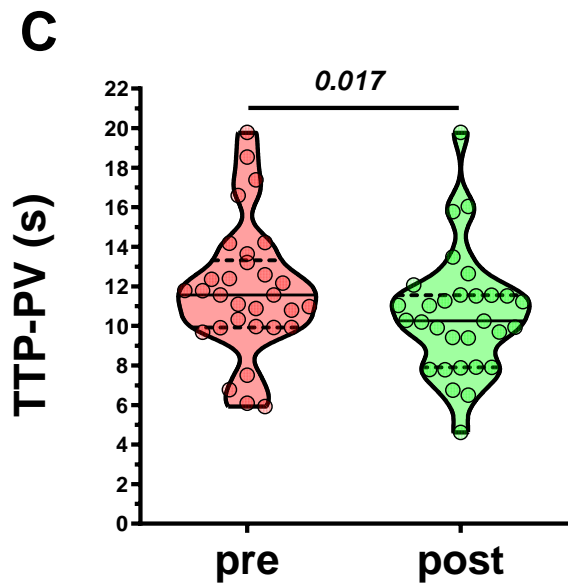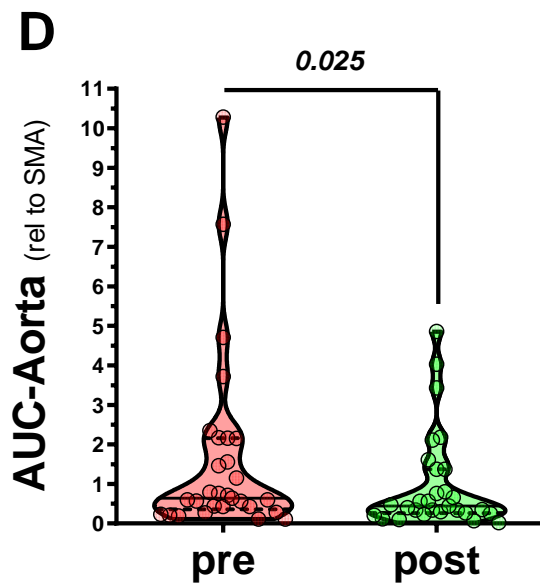

Supplement: Supplementary file 2 — Additional file 2: Figure 2. Angiographic parameters. Violine plots showing analysis of semi-quantitative NOMI score (A), peak density (PD-PV) (B) and time to peak (TTP-PV) (C) in the portal vein as well as area under the curve contrast intensity in the aorta (AU-Aorta) (E) in a subset (n = 40 for NOMI score, n = 30 for 2D-perfusion angiography-related parameters) of NOMI patients before (pre) and directly after (post) initial administration of intra-arterial prostaglandin bolus. Median (IQR) NOMI score decreased following initial prostaglandin administration (pre: 5 (4–7) points vs after: 2 (1–3) points, p < 0.001) indicating significant improvement of intestinal perfusion judged by five different categories (vessel morphology, aortal contrast reflux, contrast enhancement of the intestine, distension of the intestine and time to portal vein filling). PD-PV increased (pre: 0.665 (0.5–1.39) vs. after: 1.06 (0.39–1.7), p = 0.071) and TTP-PV decreased (pre: 11.6 (9.9–13.3) sec vs. after: 10.3 (7.9–11.6) sec, p = 0.017) following initial prostaglandin bolus, indicating better and faster portal vein filling. Reduced AUC-Aorta following prostaglandin infusion (pre: 0.64 (0.36–2.16) vs. post: 0.44 (0.26–1.37), p = 0.025) suggests less reflux into the aorta. [file 13054_2022_3962_MOESM2_ESM.pdf]

**A****L-FABP (ng/ml)**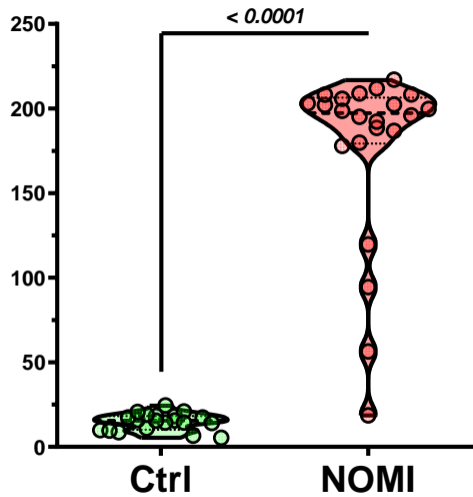**B****SM22 (pg/ml)**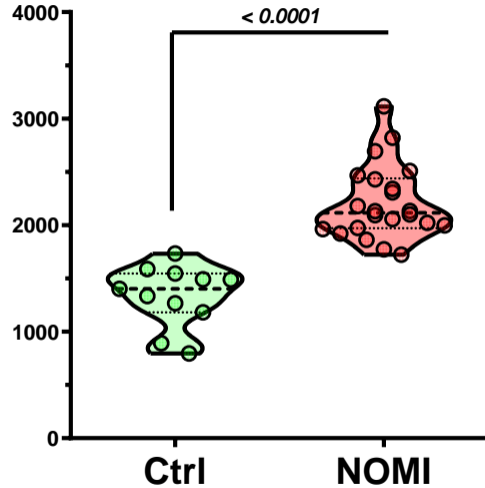**C****i-FABP (pg/ml)**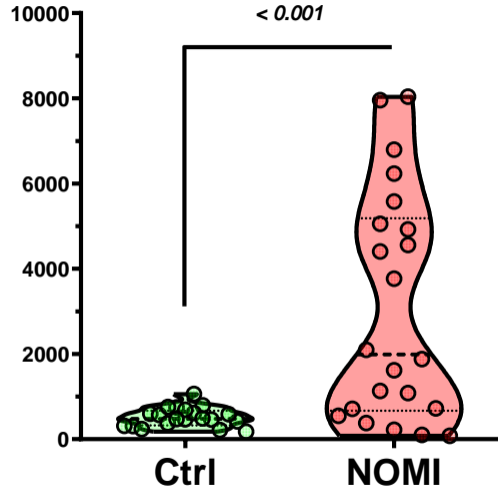

Supplement: Supplementary file 3 — Additional file 3: Figure 3. Biomarkers of intestinal ischemia. Violine plots showing analysis of L-FABP (A), i-FABP (B) and SM22 (C) in NOMI patients (n = 22) at inclusion compared to healthy controls (Ctrl) (n = 20). Median L-FABP concentrations were more than 10 times higher in NOMI patients compared to healthy controls (197 (179–206) ng/ml vs. 16 (10–18) ng/ml, p < 0.0001, A), and i-FABP was more than fourfold increased (1990 (671–5186) pg/ml vs. 479 (327–670) pg/ml, p < 0.001, B). SM22, a marker of transmural intestinal ischemia, was also significantly increased in NOMI patients (2116 (1971–2439) pg/ml vs. 1402 (1182–1546) pg/ml, p < 0.0001, C). [file 13054_2022_3962_MOESM3_ESM.pdf]
