# Supplementary material for: Predictors of response to intra-arterial vasodilatory therapy of non-occlusive mesenteric ischemia in patients with severe shock: results from a prospective observational study
Source: Crit Care. 2022 Apr 4;26:92. doi: 10.1186/s13054-022-03962-w (PMC8981621; doi:10.1186/s13054-022-03962-w)
Supplement: Supplementary file 4 — Additional file 4: Table 1. Demographic, clinical, angiographic and biochemical characteristics for non-surviving (n = 30) and surviving (n = 12) patients. 28-day mortality was the key secondary outcome. Values are presented as median (25% to 75% interquartile range) or if categorical as numbers and percentages. Peak density (PD), time to peak (TTP) and area under the curve (AUC) are in relation to the superior mesenteric artery (SMA) as reference. [file 13054_2022_3962_MOESM4_ESM.doc]

| **Suppl. Table 1:** Demographic, clinical, angiographic and biochemical characteristics for non-surviving and surviving patients | | | |
| --- | --- | --- | --- |
| **Category** | **deceased** | **alive** | **p** |
|  | (n=30) | (n=12) |  |
| Age - yr | 58 (48-70) | 67 (45-73) | 0.534 |
| Sex - no (%) |  |  | 0.379 |
| male | 17 (56.7) | 5 (41.7) |  |
| female | 13 (43.3) | 7 (58.3) |  |
| BMI - kg/m^2^ | 26.2 (21.9-28.9) | 29.2 (26.7-30.4) | **0.043** |
| Comorbidities - no (%) |  |  |  |
| Obesity | 9 (30) | 6 (50) | 0.222 |
| Hypertension | 15 (50) | 9 (75) | 0.139 |
| Diabetes | 6 (20) | 2 (16.7) | 0.804 |
| COPD | 4 (13.3) | 0 (0) | 0.184 |
| Heart insufficiency | 7 (23.3) | 4 (33.3) | 0.505 |
| CAD | 13 (43.3) | 3 (25) | 0.269 |
| CABG | 4 (13.3) | 0 (0) | 0.184 |
| PTCA | 8 (26.7) | 2 (16.7) | 0.492 |
| CKD | 13 (43.3) | 2 (16.7) | 0.103 |
| Chronic renal replacement therapy | 8 (26.7) | 2 (16.7) | 0.492 |
| Immunosuppression | 2 (6.7) | 1 (8.3) | 0.850 |
| Sepsis - no (%) | 30 (100) | 12 (100) | 1 |
| Side of infection - no (%) |  |  |  |
| pulmo | 19 (63.3) | 4 (33.3) | 0.078 |
| abdomen | 25 (83.3) | 8 (66.7) | 0.234 |
| urogenital | 5 (16.7) | 3 (25) | 0.534 |
| soft tissue | 9 (30) | 2 (16.7) | 0.375 |
| endocarditis | 1 (3.3) | 2 (16.7) | 0.130 |
| more than one | 19 (63.3) | 8 (66.7) | 0.839 |
| identified pathogen - no (%) |  |  |  |
| gram+ | 8 (26.7) | 7 (58.3) | 0.053 |
| gram- | 14 (46.7) | 6 (50) | 0.845 |
| viral | 2 (6.7) | 1 (8.3) | 0.850 |
| fungi | 8 (26.7) | 3 (25) | 0.912 |
| more than one | 8 (26.7) | 5 (41.7) | 0.342 |
| non identified | 9 (30) | 2 (16.7) | 0.375 |
| **At inclusion** |  |  |  |
| SOFA-Score - points | 17.5 (15.8-19) | 16 (12.5-18.8) | 0.221 |
| Coagulation SOFA - points | 2 (2-3) | 0.5 (0-2) | **0.003** |
| Norepinephrine - no (%) | 28 (93.3) | 12 (100) | 0.359 |
| Norepinephrine dose - µg/kg/min | 0.411 (0.253-0.631) | 0.241 (0.182-0.442) | 0.067 |
| Argipressin– no (%) | 7 (23.3) | 2 (16.7) | 0.634 |
| Dobutamine– no (%) | 4 (13.3) | 0 (0) | 0.184 |
| Invasive ventilation – no (%) | 25 (83.3) | 10 (83.3) | 1.000 |
| Oxygenationindex (PaO_2_/FiO_2_) | 202 (125-301) | 195 (158-237) | 0.765 |
| Renal replacement therapy - no (%) | 23 (76.7) | 9 (75) | 0.909 |
| Organ failure - no (%) |  |  |  |
| respiratory (PaO_2_/FiO_2_<300mmHg) | 29 (96.7) | 10 (83.3) | 0.130 |
| coagulation (Thrombocytes<100^3^/µl) | 26 (86.7) | 6 (50) | **0.012** |
| liver (Bilirubin>33μmol/l) | 23 (76.7) | 9 (75) | 0.909 |
| cardiovascular (vasopressor or inotrope) | 28 (93.3) | 12 (100) | 0.359 |
| neurological (GCS<13) | 26 (86.7) | 9 (75) | 0.359 |
| renal (Creatinine>170μmol/l) | 27 (90) | 10 (83.3) | 0.547 |
| Multi organ failure - no (%) |  |  |  |
| 2 | 0 (0) | 0 (0) |  |
| 3 | 3 (10) | 1 (8.3) | 0.868 |
| 4 | 1 (3.3) | 1 (8.8) | 0.492 |
| 5 | 10 (33.3) | 6 (50) | 0.315 |
| 6 | 16 (53.3) | 3 (25) | 0.096 |
| pH | 7.26 (7.16-7.34) | 7.34 (7.21-7.39) | 0.180 |
| Bicarbonate - mmol/l | 19 (16-22) | 20.5 (18.25-24) | 0.179 |
| Lactate - mmol/l | 9.9 (5.4-13.5) | 8.6 (4.8-12.6) | 0.409 |
| CK - U/l | 1729 (280-3106) | 3270 (507-9938) | 0.291 |
| LDH - U/l | 1305 (546-4658) | 859 (419.5-2883.3) | 0.525 |
| AST - U/l | 635 (176-2911) | 204 (117-724) | 0.877 |
| ALT - U/l | 173 (58-407) | 78 (38-234) | 0.573 |
| Bilirubin - µmol/l | 85 (28-172) | 71 (47-120) | 0.633 |
| Creatinine - µmol/l | 85 (59.3-132.3) | 100 (74-236.3) | 0.152 |
| Urea - mmol/l | 4.6 (3.6-9) | 5.8 (4.5-14.5) | 0.204 |
| Hb - g/dl | 8.8 (7.7-9.9) | 10.4 (8.9-11) | 0.097 |
| Hkt - % | 25.3 (23.4-29.9) | 30.6 (26.5-34.1) | 0.083 |
| Leucocytes - 1000/µl | 10.1 (6.3-19.8) | 14.4 (9.1-26.3) | 0.441 |
| CRP - mg/l | 108 (43-246) | 108 (48-150) | 0.139 |
| PCT - µg/l | 5.9 (1.6-27.9) | 5.5 (3.8-14.9) | 0.929 |
| Thrombocytes - 100^3^/µl | 54 (31-94) | 146 (59.8-189) | 0.054 |
| INR | 1.77 (1.25-2.13) | 1.40 (1.29-1.72) | **0.017** |
| PTT - sec | 54 (45-65.5) | 53 (42-69) | 0.931 |
| ATIII - mg/dl | 42.5 (34.5-55.8) | 57 (46.5-62.5) | 0.254 |
| i-FABP - pg/ml | 1377 (422-4795) | 5676 (1588-7978) | **0.04** |
| SM22 - pg/ml | 2132 (2031-2435) | 1968 (1760-2449) | 0.23 |
| L-FABP - ng/ml | 194 (178-207) | 201 (177-207) | 0.508 |
| **After 24 hours** |  |  |  |
| SOFA-Score - points | 17.5 (15-20) | 17 (13-18) | 0.152 |
| Coagulation SOFA - points | 2 (1.5-3.5) | 2 (1-2) | **0.032** |
| Norepinephrine - no (%) | 28 (93.3) | 10 (83.3) | 0.319 |
| Norepinephrine dose - µg/kg/min | 0.461 (0.248-0.733) | 0.155 (0.053-0.205) | **< 0.001** |
| Argipressin - no (%) | 6 (20) | 2 (16.7) | 0.804 |
| Dobutamine - no (%) | 4 (13.3) | 1 (8.3) | 0.651 |
| Invasive ventilation – no (%) | 25 (83.3) | 10 (83.3) | 1 |
| Oxygenationindex (PaO_2_/FiO_2_) | 207 (138-314) | 251 (223-280) | 0.519 |
| Organ failure - no (%) |  |  |  |
| respiratory (PaO_2_/FiO_2_<300mmHg) | 26 (86.7) | 10 (83.3) | 0.780 |
| coagulation (Thrombocytes<10^3^/µl) | 27 (93.1) | 10 (83.3) | 0.337 |
| liver (Bilirubin>33μmol/l) | 24 (80) | 9 (81.8) | 0.896 |
| cardiovascular (vasopressor or inotrope) | 28 (93.3) | 11 (91.7) | 0.850 |
| neurological (GCS<13) | 25 (83.3) | 9 (75) | 0.534 |
| renal (Creatinine>170μmol/l) | 28 (93.3) | 9 (75) | 0.097 |
| Multi organ failure - no (%) |  |  |  |
| 2 | 1 (3.3) | 1 (8.3) | 0.492 |
| 3 | 1 (3.3) | 0 (0) | 0.522 |
| 4 | 4 (13.3) | 0 (0) | 0.184 |
| 5 | 7 (23.3) | 4 (33.3) | 0.505 |
| 6 | 17 (56.7) | 6 (50) | 0.695 |
| pH | 7.32 (7.26-7.36) | 7.4 (7.34-7.44) | **0.034** |
| Bicarbonate - mmol/l | 22 (18.5-24) | 26 (22.5-27) | **0.001** |
| Lactate - mmol/l | 6.6 (3.5-12.8) | 2.4 (1.4-4.1) | **0.002** |
| CK - IU/l | 1487 (504-5875) | 1851 (722-33115) | 0.504 |
| LDH - U/l | 1397 (443-5818) | 885 (336-1345) | 0.438 |
| AST - U/l | 1065 (618-4168) | 420 (133-1218) | 0.382 |
| ALT - U/l | 320 (173-1418) | 130 (46.8-410.3) | 0.606 |
| Bilirubin - µmol/l | 85 (41-169) | 67 (35-111) | 0.369 |
| Creatinine - µmol/l | 50 (34-102) | 74.5 (48.3-144.3) | 0.235 |
| Urea - mmol/l | 3.3 (1.7-7.1) | 5.9 (2.3-13.1) | 0.123 |
| Hb - g/dl | 8.6 (7.9-9.9) | 9.1 (8.5-10.5) | 0.470 |
| Hct - % | 24.9 (22.1-29.2) | 27.2 (25.1-30.1) | 0.367 |
| Leucocytes - 1000/µl | 12.9 (6.8-19.1) | 12.8 (9-22) | 0.943 |
| CRP - mg/l | 96.5 (41.8-226) | 121 (72.5-187) | 0.764 |
| PCT - µg/l | 4.9 (1.1-22.7) | 5.8 (3.65-17.45) | 0.818 |
| Thrombocytes - 1000/µl | 59 (20.5-102) | 97 (68.3-138.8) | **0.024** |
| INR | 1.85 (1.36-2.24) | 1.46 (1.28-2.11) | 0.239 |
| PTT - sec | 53 (44.3-65.3) | 55 (40-61.3) | 0.280 |
| ATIII - mg/dl | 51 (39.5-58) | 53.5 (47-66.5) | 0.123 |
| **2D-perfusion angiography** (directly **pre**-vasodilator) |  |  |  |
| Simplified NOMI score | 5 (4-7) | 5 (3-7) | 0.876 |
| PD-PV | 0.67 (0.47-0.1.31) | 0.62 (0.57-1.88) | 0.402 |
| TTP-PV - sec | 11.57 (9.92-13.21) | 10.88 (9.91-14.18) | 0.608 |
| AUC-PV | 0.56 (0.29-0.97) | 0.75 (0.38-1.58) | 0.423 |
| PD-Aorta | 0.71 (0.28-1.55) | 1.68 (0.28-6.33) | 0.326 |
| TTP-Aorta - sec | 6.76 (6.03-9.73) | 7 (5.62-9.53) | 0.741 |
| AUC-Aorta | 0.64 (0.43-1.56) | 1.36 (0.23-5.36) | 0.380 |
| **2D perfusion angiography** (directly **post**-vasodilator**)** |  |  |  |
| Simplified NOMI score | 2.5 (1-3) | 2 (1-3.3) | 0.633 |
| PD-PV | 0.88 (0.39-1.83) | 1.37 (0.34-1.65) | 0.565 |
| TTP-PV - sec | 10.28 (7.88-12.07) | 10.24 (9.69-11.52) | 0.777 |
| AUC-PV | 0.66 (0.5-0.94) | 0.69 (0.15-0.81) | 0.316 |
| PD-Aorta | 0.48 (0.31-1.29) | 0.39 (0.24-1.26) | 0.430 |
| TTP-SMA - sec | 7.79 (6.28-9.99) | 7.37 (6.64-8.27) | 0.493 |
| AUC-Aorta | 0.47 (0.3-1.37) | 0.34 (0.20-1.13) | 0.473 |
| **Association with other outcomes** |  |  |  |
| Lactate (at 24 hrs after inclusion) |  |  |  |
| Reduction of lactate - no (%) | 21 (70) | 11 (91.7) | 0.136 |
| Δlactate - mmol/l | -1 ((-6)-(+1.2)) | -5 ((-8.6)-(-1.7)) | 0.082 |
| Δlactate - % | -18.5 ((-57.4)-(+17.9)) | -64.6 ((-75.9)-(-35.6)) | **0.019** |
| Organ dysfunction (at 24 hrs after inclusion) |  |  |  |
| Reduction of SOFA - no (%) | 8 (26.7) | 6 (50) | 0.147 |
| ΔSOFA - points | 0 ((-1)-(+2)) | -0.5 ((-1)-(+0.75)) | 0.421 |
| SOFA change - % | 0 ((-5.7)-(+12.4)) | -2.4 ((-9.3)-(+4.7)) | 0.276 |
| Vasopressor support (at 24 hrs after inclusion) |  |  |  |
| Reduction of norepinephrine - no (%) | 12 (40) | 9 (75) | **0.040** |
| ΔNE - µg/kg/min | 0.0 ((-0.11)-(+0.15)) | -0.09 ((-0.28)-(-0.00)) | 0.164 |
| ΔNE - % | 0.0 ((-20.4)-(+43.1)) | -38.0 ((-84.6)-(-8.9)) | 0.058 |

ABBREVIATIONS:

ALT – Alanine aminotransferase, ATIII – Antithrombin III, AUC – Area under the curve, AST – Aspartate aminotransferase, BMI – Body mass index, CAD – Coronary artery disease, CK – creatine kinase, CKD – Chronic kidney disease, COPD – Chronic obstructive pulmonary disease, CRP – C-reactive protein, GCS – Glasgow Coma scale, Hb – Hemoglobin, Hct – Hematocrit, i-FABP – intestinal Fatty-acid binding protein, INR – international normalized ratio, LDH – Lactate dehydrogenase, L-FABP – Liver Fatty-acid binding protein, NE – Norepinephrine, PCT – Procalcitonine, PD – Peak density, PTCA – Percutaneous transluminal coronary angioplasty, PTT – Partial thromboplastin time, PV – Portal vein, SM22 – Smooth muscle protein 22, SOFA – Sequential Organ Failure Assessment, TTP – Time to peak
